# Five key lncRNAs considered as prognostic targets for predicting pancreatic ductal adenocarcinoma

**DOI:** 10.1002/jcb.26598

**Published:** 2018-02-27

**Authors:** Jukun Song, Qiuyan Xu, Haodeng Zhang, Xinhai Yin, Chen Zhu, Ke Zhao, Jianguo Zhu

**Affiliations:** ^1^ Department of Oral and Maxillofacial Surgery Guizhou Provincial People's Hospital Guizhou China; ^2^ Department of Pathology, School of Basic Medicine Central South University Guizhou China; ^3^ Guiyang Hospital of Stomatology, Medical College Zunyi Medical College Guiyang China; ^4^ Department of Urology Guizhou Provincial People's Hospital Guizhou China

**Keywords:** lncRNAs, pancreatic ductal adenocarcinoma, weighted gene co‐expression network analysis

## Abstract

Pancreatic ductal adenocarcinoma (PDAC) has a poor prognosis, and the 5‐year survival rate was only 7.7%. To improve prognosis, a screening biomarker for early diagnosis of pancreatic cancer is in urgent need. Long non‐coding RNA (lncRNA) expression profiles as potential cancer prognostic biomarkers play critical roles in development of tumorigenesis and metastasis of cancer. However, lncRNA signatures in predicting the survival of a patient with PDAC remain unknown. In the current study, we try to identify potential lncRNA biomarkers and their prognostic values in PDAC. LncRNAs expression profiles and corresponding clinical information for 182 cases with PDAC were acquired from The Cancer Genome Atlas (TCGA). A total of 14 470 lncRNA were identified in the cohort, and 175 PDAC patients had clinical variables. We obtained 108 differential expressed lncRNA via R packages. Univariate and multivariate Cox proportional hazards regression, lasso regression was performed to screen the potential prognostic lncRNA. Five lncRNAs have been recognized to significantly correlate with OS. We established a linear prognostic model of five lncRNA (C9orf139, MIR600HG, RP5‐965G21.4, RP11‐436K8.1, and CTC‐327F10.4) and divided patients into high‐ and low‐risk group according to the prognostic index. The five lncRNAs played independent prognostic biomarkers of OS of PDAC patients and the AUC of the ROC curve for the five lncRNAs signatures prediction 5‐year survival was 0.742. In addition, targeted genes of MIR600HG, C9orf139, and CTC‐327F10.4 were explored and functional enrichment was also conducted. These results suggested that this five‐lncRNAs signature could act as potential prognostic biomarkers in the prediction of PDAC patient's survival.

## 1 INTRODUCTION

Pancreatic ductal adenocarcinoma (PDAC) remains the most common cause of cancer‐related mortality worldwide, with a 5‐year survival rate of 7.7% and average survival time of fewer than 6 months.^1^ PDAC effects 56 670 new patients each year in the United States, and is the fourth leading cause of cancer death in the United States.^2^, ^3^ The poor survival rates were due to inability to diagnosed PDAC at an early stage and to the poorly effective therapeutic methods currently available.^4^ In addition, pancreatic cancer is largely resistant to radiotherapy and chemotherapy, and little progress has been made concerning its treatment in past decades. Therefore, to decrease mortality and improve the management of PDAC, detection and risk stratification of PDAC is urgent to identify new early diagnostic biomarkers and therapeutic targets.

Long non‐coding RNA (lncRNA) is defined as RNA transcript of ≥200 bp with little or no protein‐coding capacity.^5^, ^6^, ^7^, ^8^, ^9^ At present, more and more evidence demonstrated that lncRNA played an important molecular role in apoptosis, proliferation, progression, metastasis, invasion, and relapse of the various tumor.^10^, ^11^, ^12^, ^13^, ^14^ Recent studies indicated that the dysregulated lncRNA expression profiles were associated with the development and survival in patients with various cancers, including PDCA, which uncovers the potential of lncRNA as prognostic cancer biomarker.^15^, ^16^, ^17^, ^18^, ^19^, ^20^

In an attempt to improve prognosis, a molecular screening biomarker at an early stage of pancreatic cancer is in urgent need. In this study, we aimed to explore the difference of lncRNA expression profiles between PDAC and adjacent pancreas aiming at identifying some potential lncRNA biomarkers using The Cancer Genome Atlas data (TCGA), which could help us to predict the prognosis of patients with PDAC. Moreover, these results could provide new insight into the molecular mechanism based on lncRNA for PDAC.

## 2 MATERIALS AND METHODS

### 2.1 Patient datasets

The mRNA expression and corresponding clinical information of PDAC patients were obtained from TCGA data portal (https://tcga-data.nci.nih.gov/tcga/), which was imputed on IlluminaHiSeq RNA‐Seq platform, containing 178 PDAC tissues and four adjacent non‐tumor pancreatic tissues. Both mRNA profiles data and clinical characteristics of PDAC are publicly available and open‐access. Therefore, approval by a local ethics committee was not needed.

### 2.2 lncRNA differential expression profiles

Firstly, the raw counts of PDAC mRNA expression profiles (level 3 data) were downloaded from the TCGA databases, and we acquired the lncRNA expression data by repurposing the probes in the mRNA expression profiles to lncRNA based on the annotation from the GENCODE project (http://www.gencodegenes.org).^21^ The transformed data (antisense, lincRNA, and sense_intronic) was considered as lncRNA. The RNA‐Seq data of PDCA covered 14,470 lncRNA expression profiles. Next, the differentially expressed lncRNA profiles were imputed using R/Bioconductor package of edgeR.^22^ The differentially expressed genes (DEGs) of data set with |log2 fold change| ≥ 1 and *P*‐value less than 0.05 was considered selection criteria for subsequent analysis.

### 2.3 Survival analysis and lasso regression, ROC curve

A univariate Cox model was used to calculate the association between the expression level of each lncRNA and patient's overall survival (OS). When the *P*‐values were less than 0.05, those lncRNAs were considered to be statistically significant in univariate Cox analysis. Next, multivariate Cox analysis was employed to evaluate the contribution of lncRNA as independent prognosis factors of patient survival. The backward stepwise method was conducted to further select the best model. Then, the selected lncRNAs were screened and confirmed by the Lasso regression. The lncRNA based prognosis risk score was established based on a linear combination of the expression level multiplied regression model (β) with the following formula. The Prognosis Index = (β* expression level of C9orf139) + (β* expression level of MIR600HG) + (β* expression level of RP5‐965G21.4) + (β* expression level of RP11‐436K8.1) + (β* expression level of CTC‐327F10.4).

Based on the cut‐off of the median PI, PDAC patients were divided into high‐ and low‐risk groups. The Kaplan‐Meier survival curves for the cases predicted to have low or high risk were produced. To further validate whether the prediction of the five‐lncRNA biomarkers was independent of other clinical variables, univariate and multivariate Cox regression, stratified analyses were conducted. The prognostic performance was evaluated using time‐dependent receive operating characteristic (ROC) curves within 5 years by comparing the sensitivity and specificity of the survival prediction based on the risk score. All reported *P* values were two‐sided. All analyses were performed using R/BioConductor (version 3.3.2).

### 2.4 Weighted co‐expression network construction with WGCNA and target prediction

We analyzed the incorporated network using weighted gene co‐expression network analysis (WGCNA), which can enable to describe the correlation patterns gene expression profiles. The WGCNA R package was employed to evaluate the significance of the five lncRNA and their module membership. We assessed the weighted co‐expression relation between all data set subjects in an adjacency matrix using the pairwise Pearson correlation. The appropriate soft threshold power was automatically calculated and generated as described for the standard scale‐free network. In the study, the soft threshold was set at *β* = 7 (scale free *R* ^2^ = 0.85). Following the identification of weighted correlation, characteristics of the network were presented by Cytoscape 3.4.0. We also predicted the target genes of five lncRNA by the mRNA and lncRNA network.

### 2.5 Functional enrichment analysis

The target genes of lncRNAs were selected from weighted co‐expression network. The enrichment analysis of those co‐expression protein‐coding genes was conducted using Cytoscape plug‐in ClueGO and DAVID Bioinformatics Tool (version 6.7, https://david.nciferf.gov/).^23^ Go enrichment analysis was based on the threshold of *P*‐value < 0.05 and enrichment score >1.0. Significant enrichment results were also visualized using Cytoscape software.

### 2.6 Validation of the differentially expressed lncRNA with GEO data

To verify the differentially expressed lncRNA from TCGA database, we attempt to screen the mRNA‐Sep datasets of PDCA from GEO database. To identify eligible studies, we employed the following search strategies: “pancreatic ductal adenocarcinoma” or “PDCA” or “pancreatic cancers.” The lncRNA expression level was also extracted for further analysis. The differentially expressed genes were also imputed using R package of Limma.

## 3 RESULTS

### 3.1 Differentially expression lncRNA profiles in PDAC

**Figure 1:**
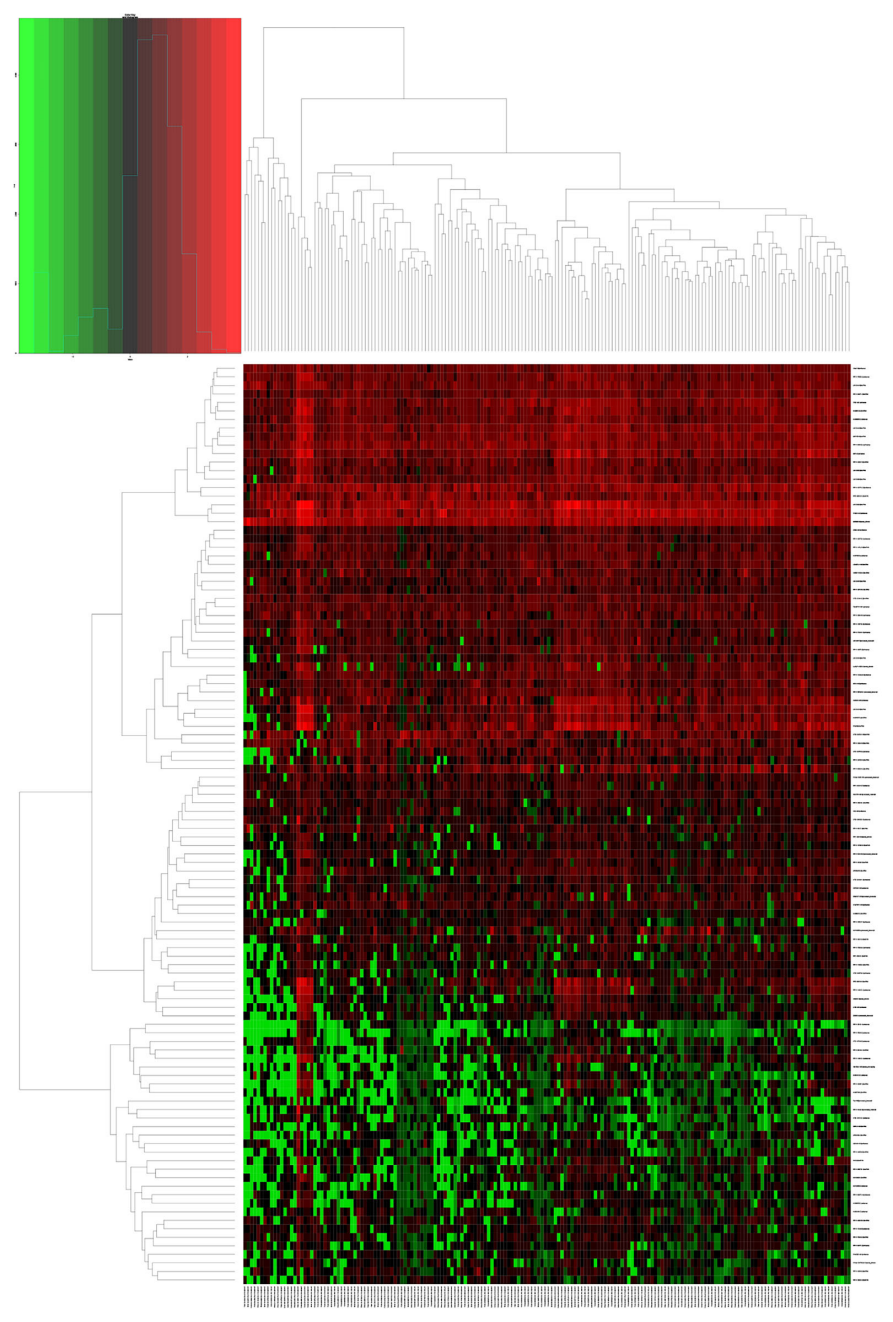
The heatmap of DElncRNAs expression in PDCA. 108 DElncRNAs were detected. Among these DElncRNAs, 3 DElncRNAs was up‐regulated genes and 105 DElncRNAs were down‐regulated genes. The color from blue to red shows a trend from low expression to high expression

The lncRNA expression profiles (level 3 data) in PDAC patients tissues (*n* = 178) compared with adjacent non‐tumor tissues (*n* = 4) was obtained from the TCGA database. A total of 109 differentially expression lncRNA was identified. Among these differentially expressed lncRNA, three lncRNAs were over‐expressed, and 106 lncRNAs were down‐expressed. Of this over‐expressed lncRNAs, three lncRNAs yielded >3‐fold increased expression including RP5‐965G21.4 and CTC‐327F10.4, 17 lncRNAs exhibited over >3‐fold decreased expression (Figure 1). The clinical data for those patients obtained from the TCGA were also available.

### 3.2 Prognostic assessment of differentially expressed lncRNA profiles and clinical characteristic

**Figure 2:**
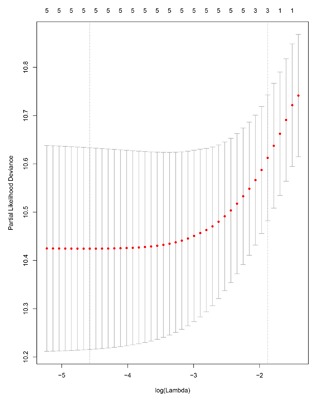
The plot of Regression coefficient diagram using the Lasso regression method

**Figure 3:**
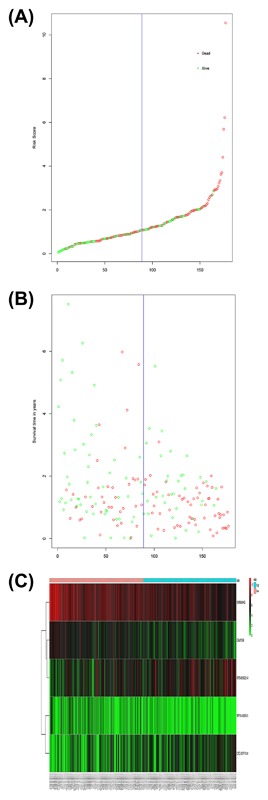
LncRNA risk score analysis of PDCA patients in TCGA. A, LncRNA risk score distribution; B, The survival status and duration of PDCA patients; C, Heatmap of the five LncRNA expression profiles in PDCA patients.

**Figure 4:**
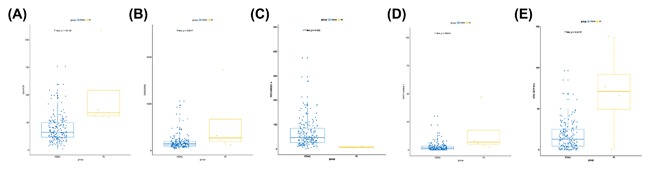
Different differential expression of the five key lncRNA between PDCA and adjunct noncancerous pancreas tissues based on TCGA data. (A) C9orf139; (B) MIR600HG; (C) RP5‐965G21.4; (D) RP11‐436K8.1; and (E) CTC‐327F10.4

**Figure 5:**
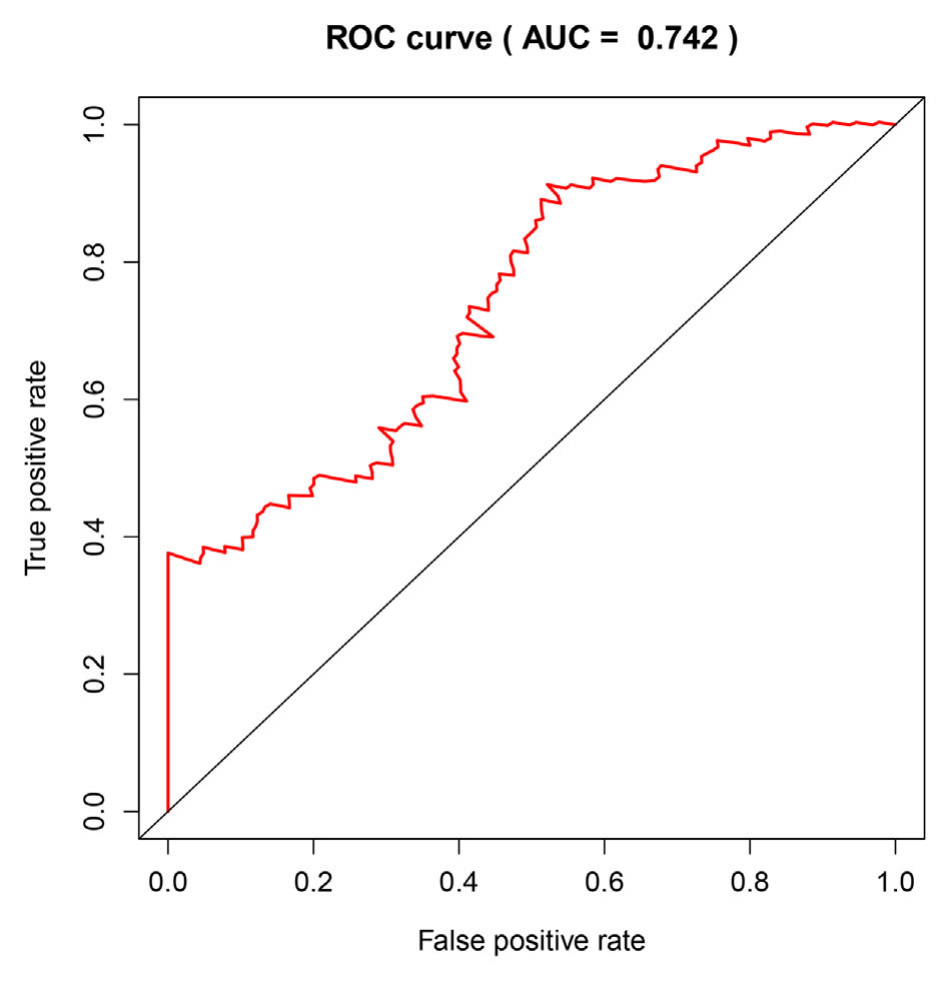
Kaplan‐Meier survival curves for overall survival outcomes according to the risk cutoff point. The *P*‐value of the log‐rank test was less than 0.01

**Figure 6:**
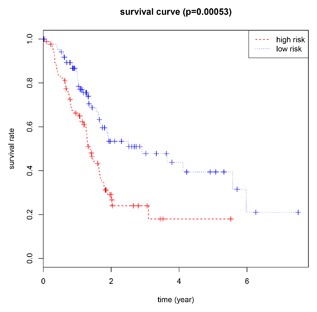
Time‐dependent ROC curves analysis for 5‐year survival prediction by the five key lncRNA

We conducted univariate Cox regression between differentially expressed lncRNA profiles and PDAC patients, and the results were shown that a total of 13 lncRNAs (MIR600HG; CC9orf139; CTC‐327F10.4; RP11‐452H21.4; RP11‐489O18.1; RP11‐436K8.1; RP5‐965G21.4; RP11‐286H15.1; RP11‐118B22.4; CTD‐2527I21.15; CH507‐513H4.5; FAM53B‐AS1; and RP11‐430C7.5) was significantly associated with OS when the *P*‐value was less than 0.05. The multivariate Cox proportional regression was applied to confirm the results above, and we found that the five lncRNA (C9orf139, MIR600HG, RP5‐965G21.4, RP11‐436K8.1, and CTC‐327F10.4) were proved to be an independent prognostic indicator for PDAC (Table 1). Next, we employed the Lasso regression to verify further variables, and the identical variables were observed (Figure 2). The prognostic index was imputed as follows: (−0.235 * expression level of C9orf139) + (−0.403 * expression level of MIR600HG) + (0.163 * expression level of RP5‐965G21.4) + (−0.187 * expression level of RP11‐436K8.1) + (0.185 * expression level of CTC‐327F10.4). We calculated the each prognostic index for each patient, and divide the patients into high or low‐risk according to the median cutoff point (Figure 3). CTC‐327F10.4 and RP5‐965G21.4 were high expressions in both groups, while C9orf139 and MIR600HG, RP11‐436K8.1 were low expressions in PDAC group in both groups (Figure 4). Kaplan‐Meier curves for the high‐ and low‐risk groups are shown in Figure 5. Patients with high‐risk score showed poorer OS than patients with those who have low risk score (median OS of 15.8 months vs 14.2 months). The HR of the risk score produced by the univariate Cox proportional hazards regression method was 2.11 (95%CI, 1.37‐3.24) and multivariate Cox proportional hazards regression method also show the consistent result (HR = 1.91, 95%CI: 1.22‐2.98) adjusted for the clinical covariate. We employed time‐dependent ROC curves to evaluate the prognostic power of five‐lncRNAs biomarkers. The AUC for the six‐lncRNA biomarkers prognostic model was 0.727 at 5 years of OS (Figure 6).

**Table 1:** Five lncRNA significantly correlated with overall survival

Gene name	Ensembel ID	Chromosome	*P*‐value	Hazard ratio	Coefficient
C9orf139	ENSG00000180539	Chromosome 9: 137,027,464‐137,037,957	0.000113	0.628473	−0.4645
MIR600HG	ENSG00000236901	Chromosome 9: 123,109,494‐123,115,477	4.74E‐06	0.492488	−0.7083
RP5‐965G21.4	ENSG00000274414	Chromosome 20: 25,239,007‐25,245,229	0.007241	1.253022	0.22556
RP11‐436K8.1	ENSG00000231252	Chromosome 1: 60,659,631‐60,867,998	0.738693	0.00566	−0.3029
CTC‐327F10.4	ENSG00000251320	Chromosome 5: 147,887,112‐147,887,704	0.00041	1.277125	0.24461

Subsequently, the univariate Cox regression between clinical features and PDAC was performed to confirm the prognostic significance of the clinical characteristics. After analysis, clinical covariates of N stage and Person neoplasm cancer status, Primary therapy outcome success were significantly correlated with OS. However, clinical covariates of gender, tumor grade, T stage, M stage, pathologic disease stage, cancer status, new tumor event after initial treatment, and primary therapy outcome success were not correlated with OS (Table 2).

**Table 2:** Univariate and multivariate Cox regression analysis in PDAC cohort

	Univariate analysis		Multivariate analysis	
Variables	HR (95%CI)	*P*‐value	HR (95%CI)	*P*‐value
Gender (male/female)	1.273 (0.837‐1.935)	0.260	1.143 (0.584‐2.263)	0.697
Age (>50/≤50)	1.185 (0.612‐2.294)	0.614	1.327 (0.853‐2.067)	0.673
Tumor grade (I‐II/III‐IV)	1.417 (0.915‐2.194)	0.118		
T stage (T1‐T2/T3‐T4)	1.844 (0.951‐3.573)	0.070		
N stage (N1/N0)	2.190 (1.288‐3.722)	0.004		
M stage (M1/M0)	0.934 (0.755‐1.155)	0.529		
AJCC stage (I‐II/III‐IV)	0.799 (0.252‐2.536)	0.703	0.736 (0.223‐2.425)	0.614
New tumor event after initial treatment (yes/no)	1.399 (0.889‐2.202)	0.147	1.072 (0.775‐1.483)	0.673
Person neoplasm cancer status (with tumor/tumor free)	3.542 (1.886‐6.652)	0.000	1.139 (0.782‐1.658)	0.498
Primary therapy outcome success (SD + PD/CR + PR)	0.472 (0.272‐0.818)	0.008	0.484 (0.259‐0.903)	0.025
Five lncRNA risk score	1.976 (1.275‐3.064)	0.002	2.031 (1.300‐3.175)	0.002

**Figure 7:**
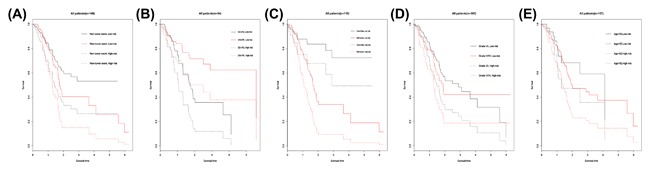
Kaplan‐Meier survival curves in stratified analysis according to different clinical features. (A) New tumor event after initial treatment, (B) Primary therapy outcome success, (C) Cancer status, (D) Grade, and (E) Age

**Figure 8:**
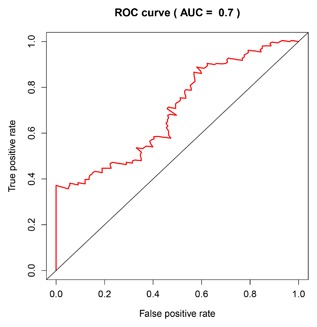
Prediction value of risk scores for early stage (I‐II)

In the meantime, the prognostic value of different clinicopathological characteristics was also examined. The K‐M curves revealed that tumor status and grade classification, new tumor event after initial treatment, primary therapy outcome success could manifest the outcome between high‐risk and low‐risk groups (Figure 7). Since patients with the early tumor stage may benefit significantly from a prognostic biomarker signature, we also assess the prognostic power of the five‐lncRNAs in stage I and II PDAC tumors (*n* = 160). The prediction also demonstrated good performance on early tumors (AUC = 0.700, Figure 8).

### 3.3 Functional evaluation of the five‐lncRNA by WGCNA

**Figure 9:**
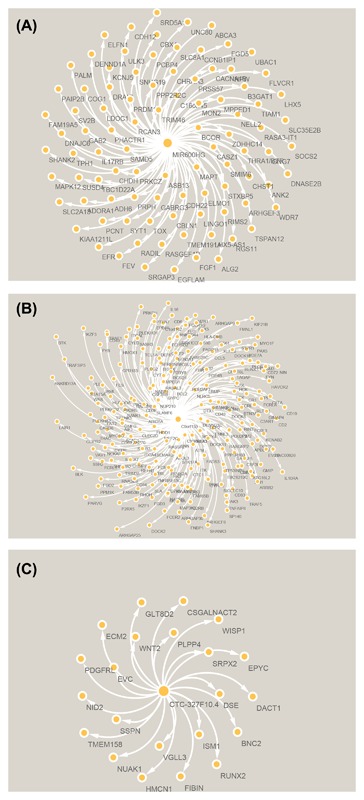
The network of lncRNA MIR600HG (A), C9orf139 (B), and CTC‐327F10.4 (C) with co‐expression genes by WGCNA

The biological functions of lncRNAs were still largely unknown. Therefore, to explore the target prediction of five key lncRNAs, we employed the Weighted Co‐expression network construction to examine the functions. The relevant genes of key lncRNA C9orf139 are AIF1L, NLRC5, FMNL3, P2RY8, SP140, LRRK1, FAM65B, SASH3, KIF21B, IKZF3, TRAF1, WAS, BANK1, GLIPR2, PLEKHO2, FGD2, CD69, PARVG, MS4A4A, PDE4B, CD6, CIITA, TNFAIP8, FCRL2, PLEKHA2, and LRMP (Figure 9A). The targeted genes of key lncRNA CTC‐327F10.4 are DCBLD1, DSE, PDGFRL, HMCN1, NUAK1, NID2, ISM1, EVC, SRPX2, ECM2, DACT1, VGLL3, CSGALNACT2, BNC2, WNT2, TMEM158, SSPN, RUNX2, EPYC, FIBIN, PLPP4, WISP1, and GLT8D2 (Figure 9B). The targeted genes of key lncRNA MIR600HG are TMEFF2, MPPED1, RASGEF1B, SLC8A1, NELL2, CASZ1, TSPAN12, SOCS2, CDH22, UNC80, ZDHHC14, FLVCR1, FAM19A5, FEV, LINGO1, SMIM6, PRKCZ, COG1, ASB13, FGD5, RIMS2, ARHGEF3, PCNT, MAPK12, FGF1, TIAM1, EFR3B, DRAIC, PALM, SHANK2, BCOR, CHST1, GNG7, RASA3‐IT1, LHX5‐AS1, SLC35E2B, THRA1/BTR, MON2, SYT1, DNAJC6, EGFLAM, LDOC1, TMEM191A, ULK3, PCBP4, PPP2R2C, CACNA1B, ELMO1, PRDM16, KIAA1211L, CCNB1IP1, CHRNA3, TRIM46, KCNJ5, RCAN3, SAMD5, ELFN1, SUSD4, IL17RB, UBAC1, CBX4, GABRG3, ALG2, C16orf45, ADH6, DENND1A, GAB2, SRD5A1, PAIP2B, TPH1, CBLN1, ABCA3, SV2B, SNHG19, ANK2, SRGAP3, PRSS57, WDR7, NPW, PHACTR1, CHDH, SLC2A13, DNASE2B, ADORA1, STXBP5, RGS11, TOX, LHX5, B3GAT1, CDH12, TBC1D22A, PRPH, RADIL, and MAPT (Figure 9C).

**Figure 10:**
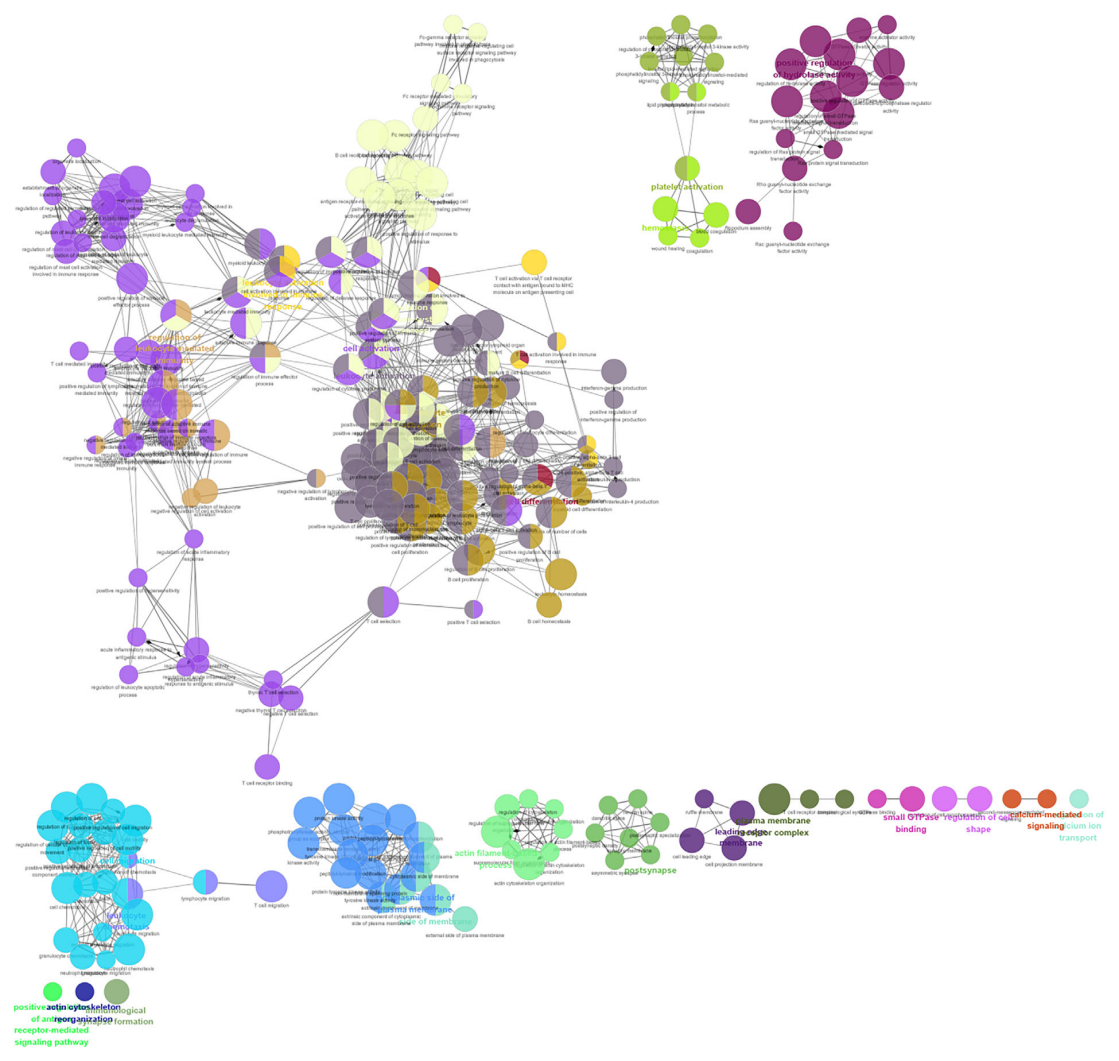
GO terms shows as an interaction network using Cytoscape plug‐in ClueGO

**Figure 11:**
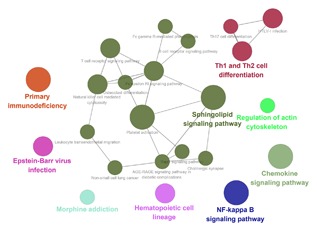
KEGG pathways displayed as an interaction network Cytoscape plug‐in ClueGO

A total of 333 target genes were identified as potentially regulated by the lncRNA MIR600HG, CTC‐327F10.4, and C9orf139 in the co‐expression network. The enrichment analysis was conducted to describe the biological function of the target of lncRNA biomarkers. It revealed enrichment of 301 gene ontology categories. The enrichment biological process was shown in Figure 10. A total of 21 KEGG pathways were enriched by the target genes (Figure 11), including Primary immunodeficiency, natural killer cell mediated cytotoxicity, Rap1 signaling pathway, and regulation of actin cytoskeleton.

### 3.4 Validation of the five lncRNA expression with GEO database

Five studies were considered eligible from GEO, including GSE15471, GSE28735, GSE32676, GSE41368, and GSE71989. However, only two key lncRNA expression (C9orf139 and MIR600HG) could find in two datasets (GSE28735 and GSE41368).

## 4 DISCUSSION

Pancreatic ductal adenocarcinoma (PDAC) remains one of the most aggressive and lethal commonly diagnosed cancers worldwide by complex molecular and cellular heterogeneity.^24^ In the past, great efforts have been made to provide novel insights into the molecular mechanisms underlying PDCA, but the focus has been on protein‐coding genes or miRNA.^25^, ^26^, ^27^, ^28^, ^29^ PDAC is the most common form of pancreatic cancer and its incidence is rising every year.^30^ Therefore, understanding of PDAC biology may provide clinicians with new tools that can be used for the treatment of this disease. Up to date, there is increasing evidence that lncRNAs are a novel class of non‐protein‐coding transcripts that have been reported in cancer biogenesis and prognosis. Integrative genomic studies have demonstrated that the roles of lncRNA have attracted considerable attention. Many potential and valuable lncRNA are needed to be identified to improve the clinical outcome of PDAC patients. The dysfunction of lncRNA can exist in various cancers and are significantly correlated with prognosis of cancers.^15^, ^16^, ^31^, ^32^, ^33^ However, none specific biomarkers have been found to display the therapeutic efficiency, and prognosis factor is of great importance for the treatment of PDAC patients.

Recently several studies have reported that long non‐coding RNA as early prediction and diagnostic biomarkers of PDAC had been identified. Experimental evidence revealed that the upregulated MIR31HG acts as an oncogenic lncRNA that promotes tumor progression.^34^ Based on the microarray data, Li et al identified 5250 differentially expressed lncRNA in PDAC, including 1881 upregulated lncRNAs and downregulated 3369 lncRNAs. The high expression level of lncRNA BC008363 had significantly better survival rates.^35^ A more recent study reported that the lncRNA HOTTIP/HOXA13 played a potential therapeutic target and molecular biomarker for PDAC.^36^ Therefore, in an attempt to decrease mortality and improve prognosis of PDCA, a molecular screening biomarker at an early stage of pancreatic cancer is in urgent need. In this study, we aimed to explore the difference of genes expression between PDAC patients and adjacent non‐tumor pancreatic tissues aiming at identifying some potential lncRNA biomarkers using a database of The Cancer Genome Atlas. The differentially expressed lncRNAs were screened, and we conducted univariate and multivariate Cox analysis to verify these lncRNAs for prediction of PDAC prognosis further. Lastly, we have recognized five lncRNA consisting of C9orf139, MIR600HG, RP5‐965G21.4, RP11‐436K8.1, and CTC‐327F10.4. It was then validated to be an independent prognosis predictor for patients with PDAC. The AUC of the ROC curve for the five lncRNA signature predicting 3‐year survival was 0.742. The five lncRNA signatures have a good performance for survival prediction. The result also demonstrated that the five lncRNA was independent of other clinical factors in PDAC. Among these lncRNAs, low expression (CTD‐2527I21.15 and CH507‐513H4.5) and overexpression of lncRNA (MIR600HG and CC9orf139, RP11‐489O18.1) were associated with poor prognosis in patients with PDAC. Although the target gene of these lncRNAs has not been previously studied in cancers, we figured out that these lncRNAs may be involved PDAC tumorigenesis and many studies are needed to validate the findings in the future. Through Weighted Co‐expression network using WGCNA, we found that lncRNA MIR600HG, CTC‐327F10.4, and C9orf139 are correlated with 333 protein‐coding genes. Using the enrichment and functional analysis of Cytoscape plug‐in ClueGO and DAVID Bioinformatics Tool, we found that the Gene ontology of targeted genes were involved in immunological synapse formation, regulation of cell morphogenesis, and lymphocyte activation involved in immune response. Aberrant activation of Primary immunodeficiency, natural killer cell mediated cytotoxicity, Rap1 signaling pathway, and regulation of actin cytoskeleton pathway may inhibit tumor cells growth and proliferation, progression. To our knowledge, the five‐lncRNA biomarkers have not been previously studied, and further understanding the function of the five lncRNAs will help the clinician to early diagnose the patients and bring some clinical indications in the development of novel prognostic factors in PDAC.

Several limitations of the current study should be considered. First, the prognostic power of five lncRNAs signature was only analyzed and validated in the TCGA data set, and no other PDAC lncRNAs expression profile can be used for further validation. Second, the TCGA data was from a single central source and ethics of population in TCGA database was mainly confined to white and black, the findings in the work cannot extrapolated to other ethics. Third, no experimental data on the potential mechanisms of the lncRNA have been reported, and further experimental studies on these aspects can enhance our understanding of the functional role in PDAC.

In this work, our findings demonstrated that the five lncRNAs molecular biomarkers were identified to predict 5‐year survival in patients with PDAC, which could become a novel prognostic indicator for predicting the clinical outcome. However, the biological function of these five lncRNAs needs to be further validated with more experiment.

## References

[1] Vogelzang NJ , Benowitz SI , Adams S , et al. Clinical cancer advances 2011: Annual Report on Progress Against Cancer from the American Society of Clinical Oncology. J Clin Oncol. 2012; 30:88–109. 22147736 10.1200/JCO.2011.40.1919

[2] Chen W , Zheng R , Zhang S , Zhao P , Zeng H , Zou X . Report of cancer incidence and mortality in China, 2010. Ann Transl Med. 2014; 2:61. 25333036 10.3978/j.issn.2305-5839.2014.04.05 PMC4202458

[3] Siegel RL , Miller KD , Jemal A . Cancer statistics, 2017. CA Cancer J Clin. 2017; 67:7–30. 28055103 10.3322/caac.21387

[4] Jones S , Zhang X , Parsons DW , et al. Core signaling pathways in human pancreatic cancers revealed by global genomic analyses. Science. 2008; 321:1801–1806. 18772397 10.1126/science.1164368 PMC2848990

[5] Gloss BS , Dinger ME . The specificity of long noncoding RNA expression. Biochim Biophys Acta. 2016; 1859:16–22. 26297315 10.1016/j.bbagrm.2015.08.005

[6] Li Y , Wang X . Role of long noncoding RNAs in malignant disease (review). Mol Med Rep. 2016; 13:1463–1469. 26708950 10.3892/mmr.2015.4711

[7] Heery R , Finn SP , Cuffe S , Gray SG . Long non‐coding RNAs: key regulators of epithelial‐mesenchymal transition, tumour drug resistance and cancer stem cells. Cancers (Basel). 2017; 9. 10.3390/cancers9040038 PMC5406713 28430163

[8] Wang KC , Chang HY . Molecular mechanisms of long noncoding RNAs. Mol Cell. 2011; 43:904–914. 21925379 10.1016/j.molcel.2011.08.018 PMC3199020

[9] Zhang F , Zhang L , Zhang C . Long noncoding RNAs and tumorigenesis: genetic associations, molecular mechanisms, and therapeutic strategies. Tumour Biol. 2016; 37:163–175. 26586396 10.1007/s13277-015-4445-4

[10] Jin L , Fu H , Quan J , et al. Overexpression of long non‐coding RNA differentiation antagonizing non‐protein coding RNA inhibits the proliferation, migration and invasion and promotes apoptosis of renal cell carcinoma. Mol Med Rep. 2017; 16:4463–4468. 28765964 10.3892/mmr.2017.7135

[11] Li D , Li H , Yang Y , Kang L . Long noncoding RNA urothelial carcinoma associated 1 promotes the proliferation and metastasis of human lung tumor cells by regulating microRNA‐144. Oncol Res. 2017;1 https://doi.org/10.3727/096504017X15009792179602. [Epub ahead of print]. 10.3727/096504017X15009792179602 PMC7844600 28762326

[12] Li S , Yang J , Xia Y , Fan Q , Yang KP . LncRNA NEAT1 promotes proliferation and invasion via targeting miR‐181a‐5p in non‐small cell lung cancer. Oncol Res. 2017 https://doi.org/10.3727/096504017X15009404458675. [Epub ahead of print]. 10.3727/096504017X15009404458675 PMC7844613 28762332

[13] Lu S , Zhou J , Sun Y , et al. The noncoding RNA HOXD‐AS1 is a critical regulator of the metastasis and apoptosis phenotype in human hepatocellular carcinoma. Mol Cancer. 2017; 16:125. 28724429 10.1186/s12943-017-0676-x PMC5518122

[14] Zhou M , Xu W , Yue X , et al. Relapse‐related long non‐coding RNA signature to improve prognosis prediction of lung adenocarcinoma. Oncotarget. 2016; 7:29720–29738. 27105492 10.18632/oncotarget.8825 PMC5045428

[15] Huang C , Yu W , Wang Q , et al. Increased expression of the lncRNA PVT1 is associated with poor prognosis in pancreatic cancer patients. Minerva Med. 2015; 106:143–149. 25668599

[16] Fu XL , Liu DJ , Yan TT , et al. Analysis of long non‐coding RNA expression profiles in pancreatic ductal adenocarcinoma. Sci Rep. 2016; 6:33535. 27628540 10.1038/srep33535 PMC5024322

[17] Han D , Gao X , Wang M , et al. Long noncoding RNA H19 indicates a poor prognosis of colorectal cancer and promotes tumor growth by recruiting and binding to eIF4A3. Oncotarget. 2016; 7:22159–22173. 26989025 10.18632/oncotarget.8063 PMC5008352

[18] Li C , Zhou L , He J , Fang XQ , Zhu SW , Xiong MM . Increased long noncoding RNA SNHG20 predicts poor prognosis in colorectal cancer. BMC Cancer. 2016; 16:655. 27543107 10.1186/s12885-016-2719-x PMC4992210

[19] Liu T , Zhang X , Yang YM , Du LT , Wang CX . Increased expression of the long noncoding RNA CRNDE‐h indicates a poor prognosis in colorectal cancer, and is positively correlated with IRX5 mRNA expression. Onco Targets Ther. 2016; 9:1437–1448. 27042112 10.2147/OTT.S98268 PMC4795576

[20] Yong S , Yabin Y , Bing Z , et al. Reciprocal regulation of DGCR5 and miR‐320a affects the cellular malignant phenotype and 5‐FU response in pancreatic ductal adenocarcinoma. Oncotarget. 2017; 8:90868–90878. 29207609 10.18632/oncotarget.18377 PMC5710890

[21] Derrien T , Johnson R , Bussotti G , et al. The GENCODE v7 catalog of human long noncoding RNAs: analysis of their gene structure, evolution, and expression. Genome Res. 2012; 22:1775–1789. 22955988 10.1101/gr.132159.111 PMC3431493

[22] Robinson MD , McCarthy DJ , Smyth GK . EdgeR: a Bioconductor package for differential expression analysis of digital gene expression data. Bioinformatics. 2010; 26:139–140. 19910308 10.1093/bioinformatics/btp616 PMC2796818

[23] Huang da W , Sherman BT , Lempicki RA . Bioinformatics enrichment tools: paths toward the comprehensive functional analysis of large gene lists. Nucleic Acids Res. 2009; 37:1–13. 19033363 10.1093/nar/gkn923 PMC2615629

[24] Waddell N , Pajic M , Patch AM , et al. Whole genomes redefine the mutational landscape of pancreatic cancer. Nature. 2015; 518:495–501. 25719666 10.1038/nature14169 PMC4523082

[25] Calatayud D , Dehlendorff C , Boisen MK , et al. Tissue MicroRNA profiles as diagnostic and prognostic biomarkers in patients with resectable pancreatic ductal adenocarcinoma and periampullary cancers. Biomark Res. 2017; 5:8. 28239461 10.1186/s40364-017-0087-6 PMC5320745

[26] Karamitopoulou E , Haemmig S , Baumgartner U , Schlup C , Wartenberg M , Vassella E . MicroRNA dysregulation in the tumor microenvironment influences the phenotype of pancreatic cancer. Mod Pathol. 2017; 30:1116–1125. 28548126 10.1038/modpathol.2017.35

[27] Lu H , Niu F , Liu F , Gao J , Sun Y , Zhao X . Elevated glypican‐1 expression is associated with an unfavorable prognosis in pancreatic ductal adenocarcinoma. Cancer Med. 2017; 6:1181–1191. 28440066 10.1002/cam4.1064 PMC5463070

[28] Mikamori M , Yamada D , Eguchi H , et al. MicroRNA‐155 controls exosome synthesis and promotes gemcitabine resistance in pancreatic ductal adenocarcinoma. Sci Rep. 2017; 7:42339. 28198398 10.1038/srep42339 PMC5309735

[29] Yu DL , Zhang T , Wu K , et al. MicroRNA‐448 suppresses metastasis of pancreatic ductal adenocarcinoma through targeting JAK1/STAT3 pathway. Oncol Rep. 2017; 38:1075–1082. 28677798 10.3892/or.2017.5781

[30] Dunne RF , Hezel AF . Genetics and biology of pancreatic ductal adenocarcinoma. Hematol Oncol Clin North Am. 2015; 29:595–608. 26226899 10.1016/j.hoc.2015.04.003 PMC5697145

[31] Chen X , You ZH , Yan GY , Gong DW . IRWRLDA: improved random walk with restart for lncRNA‐disease association prediction. Oncotarget. 2016; 7:57919–57931. 27517318 10.18632/oncotarget.11141 PMC5295400

[32] Zhang XQ , Sun S , Lam KF , et al. A long non‐coding RNA signature in glioblastoma multiforme predicts survival. Neurobiol Dis. 2013; 58:123–131. 23726844 10.1016/j.nbd.2013.05.011

[33] Zhou M , Zhao H , Wang Z , et al. Identification and validation of potential prognostic lncRNA biomarkers for predicting survival in patients with multiple myeloma. J Exp Clin Cancer Res. 2015; 34:102. 26362431 10.1186/s13046-015-0219-5 PMC4567800

[34] Yang H , Liu P , Zhang J , et al. Long noncoding RNA MIR31HG exhibits oncogenic property in pancreatic ductal adenocarcinoma and is negatively regulated by miR‐193b. Oncogene. 2016; 35:3647–3657. 26549028 10.1038/onc.2015.430 PMC4947634

[35] Li J , Liu D , Hua R , et al. Long non‐coding RNAs expressed in pancreatic ductal adenocarcinoma and lncRNA BC008363 an independent prognostic factor in PDAC. Pancreatology. 2014; 14:385–390. 25200694 10.1016/j.pan.2014.07.013

[36] Tong YS , Wang XW , Zhou XL , et al. Identification of the long non‐coding RNA POU3F3 in plasma as a novel biomarker for diagnosis of esophageal squamous cell carcinoma. Mol Cancer. 2015; 14:3. 25608466 10.1186/1476-4598-14-3 PMC4631113

